# Genome-Wide Identification of DUF668 Gene Family and Expression Analysis under *F. solani*, Chilling, and Waterlogging Stresses in *Zingiber officinale*

**DOI:** 10.3390/ijms25020929

**Published:** 2024-01-11

**Authors:** Shuo Han, Xiaowen Han, Chuandong Qi, Fengling Guo, Junliang Yin, Yiqing Liu, Yongxing Zhu

**Affiliations:** 1 Industrial Crops Institute of Hubei Academy of Agricultural Sciences, Key Laboratory of Vegetable Ecological Cultivation on Highland, Ministry of Agriculture and Rural Affairs, Wuhan 430064, China; 2022710815@yangtzeu.edu.cn (S.H.); 2022710817@yangtzeu.edu.cn (X.H.); qichuandong@hbaas.com (C.Q.); 2 MARA Key Laboratory of Sustainable Crop Production in the Middle Reaches of the Yangtze River, Co-Construction by Ministry and Province, College of Agriculture, Yangtze University, Jingzhou 434025, China; liung906@163.com (Y.L.); yongxingzhu@yangtzeu.edu.cn (Y.Z.)

**Keywords:** ginger, DUF668, gene expression, biotic/abiotic stress

## Abstract

The domains of unknown function (DUF) superfamilies contain proteins with conserved amino acid sequences without known functions. Among them, DUF668 was indicated widely involving the stress response of plants. However, understanding ZoDUF668 is still lacking. Here, 12 *ZoDUF668* genes were identified in ginger by the bioinformatics method and unevenly distributed on six chromosomes. Conserved domain analysis showed that members of the same subfamily had similar conserved motifs and gene structures. The promoter region of *ZoDUF668s* contained the light, plant hormone and stress-responsive elements. The prediction of miRNA targeting relationship showed that nine ginger miRNAs targeted four *ZoDUF668* genes through cleavage. The expression patterns of 12 *ZoDUF668* genes under biotic and abiotic stress were analyzed using RT-qPCR. The results showed that the expression of seven *ZoDUF668* genes was significantly downregulated under *Fusarium solani* infection, six *ZoDUF668* genes were upregulated under cold stress, and five *ZoDUF668* genes were upregulated under waterlogging stress. These results indicate that the *ZoDUF668* gene has different expression patterns under different stress conditions. This study provides excellent candidate genes and provides a reference for stress-resistance research in ginger.

## 1. Introduction

*Zingiber officinale* Roscoe, a perennial herb belonging to Zingiberaceae, is widely cultivated for its distinctive edible and medicinal values [1]. With the increase in market demand, the planting scale of ginger has gradually expanded, and the problems restricting the yield and quality of ginger have become increasingly prominent. Among them, the factors that affect the yield and quality of ginger mainly include various biotic and abiotic stresses, including ginger blast, stem rot, ginger borer, drought, and waterlogging [2]. Cultivating stress-tolerant varieties is currently the most economical and effective way to reduce losses. Therefore, identifying and excavating genes related to stress resistance and screening resistant ginger germplasm resources are of great significance for ginger stress-resistance breeding.

The domains of unknown function protein families (DUFs) refer to a class of protein families with unknown functional domains [3,4]. At present, the Pfam database has included 6565 DUFs or unspecified protein families (UPFs), accounting for approximately 34% of the entire protein family in the database [5]. The DUFs not only participate in plant growth and development, but also play a positive regulatory role in plant response to biotic/abiotic stresses [3,6]. For example, Yuan et al. [7] found that a protein STS1 containing the DUF726 domain plays a crucial role in the development of rice tapetum and pollen wall formation. *AtDUF569* positively regulates stem growth and negatively regulates root growth in *Arabidopsis thaliana* under the stress of nitric oxide [8]. In *Gossypium hirsutum*, the DUF1117 protein encoded by *GhRDUF4D* increased its expression level after inoculation with *Verticillium dahlia* [6]. In *Triticum aestivum*, after *TaDUF966-9B* was silenced, its leaves exhibited severe leaf curling symptoms under salt stress, indicating that *TaDUF966s* played a positive regulatory role in wheat’s response to salt stress [9].

DUF668 is one of the highly conserved plant-specific transcription factors, characterized by a conserved domain containing 29 amino acids, which are widely present in monocotyledonous and dicotyledonous plants [10]. This domain was initially discovered in *Arabidopsis thaliana* and is located at the C-terminus of the *A. thaliana* PSI protein, which has a promoting effect on plant growth [11]. *DUF668* genes have shown the potential importance of participating in stress resistance in plants [3]. In *Glycine max*, *GmDUF668-8*, *GmDUF668-20*, *GmDUF668-30* respond to salt stress by upregulating their expression levels [12]. In *T. aestivum*, *TaDUF668-26* and *TaDUF668-27* participate in the infection process of *Fusarium graminearum* and *Puccinia striiformis* [10]. In *Ipomoea batatas*, *IbDUF668-6*, *IbDUF668-7*, *IbDUF668-11* and *IbDUF668-13* could respond to salt and drought stresses [13].

So far, DUF668 family members have been identified and analyzed in rice [3], cotton [6], sweet potato [13], soybean [12] and wheat [10], and their stress response patterns have been studied. However, there is still a lack of systematic understanding of DUF688 family members in ginger. Therefore, this study systematically identified the members of the DUF668 family in ginger and analyzed the expression characteristics of this gene family under various stress conditions, aiming to preliminarily understand the function of the *DUF668* gene in ginger and to provide a theoretical basis for resistance breeding of ginger.

## 2. Results

### 2.1. Identification of ZoDUF668s

**Figure 1:**
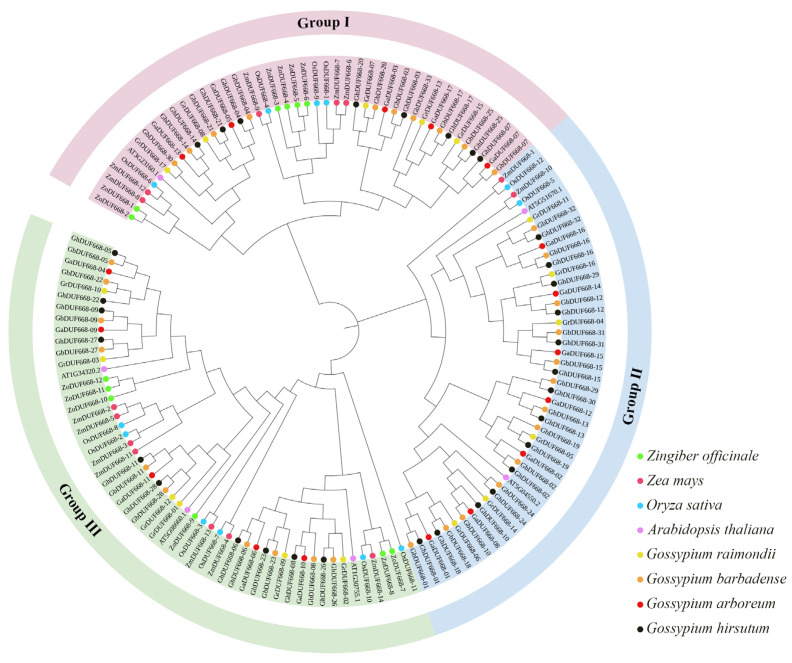
Phylogenetic tree of DUF668 proteins.

Through BLASTp alignment and domain analysis using Pfam database, 12 ginger ZoDUF668 members were identified. According to the evolutionary relationship of the phylogenetic tree, they were designated as *ZoDUF668-1*~*ZoDUF668-12*. To determine the evolutionary relationship between *ZoDUF668s* and other plants, a phylogenetic tree was constructed using 143 DUF668 members, including *Z. officinale*, *A. thaliana*, *O. sativa*, *Z. mays*, and Gossypium. The results showed that the DUF668 protein of five species can be divided into three groups (Group I, II, and III). Group III has the highest number of DUF668, containing 53 genes, including six *ZoDUF668s*, followed by Group II and Group I, containing 46 and 44 genes, respectively, including zero and six *ZoDUF68s*, respectively (Figure 1).

### 2.2. Chromosomal Distribution of ZoDUF668s

**Figure 2:**
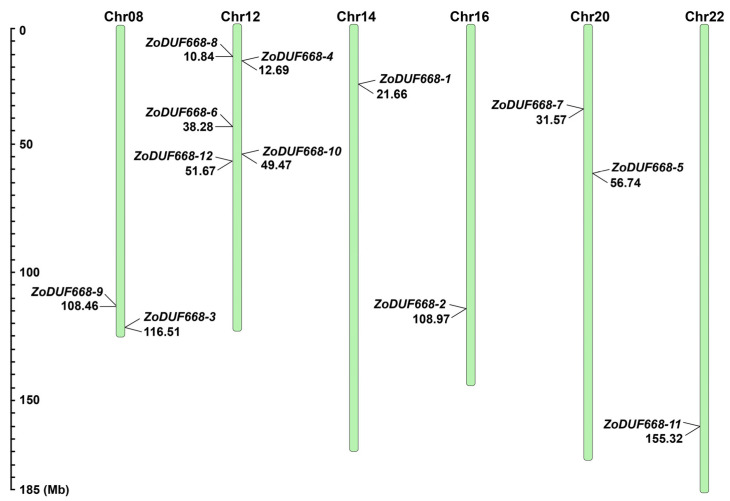
Chromosomal distribution of *ZoDUF668s*. Chr stands for chromosome. The ruler on the left represents the length of chromosomes (Mb).

As can be seen in Figure 2, 12 *ZoDUF668* genes are unevenly distributed on six chromosomes. Among them, Chr12 (Chromosome 12) has the highest number of genes, including five *ZoDUF668* genes, followed by Chr8 and Chr20, both containing two *ZoDUF668* genes, and the remaining chromosomes only contain one *ZoDUF668* gene.

### 2.3. Characterization Analysis of ZoDUF668 Proteins Sequence

As shown in Table 1, the average length of the ZoDUF668 protein is 674.75 aa, with a distribution range of 471–1428 aa. The average molecular weight of the protein is 75.27 kDa, and the distribution range is 52.91–156.83 kDa. Among them, ZoDUF668-7 has the largest molecular weight of 156.83 kDa, and the remaining proteins are all less than 100 kDa. The ZoDUF668 isoelectric point ranges from 6.64 to 9.68, and 10 ZoDUF668 proteins are predicted to be alkaline proteins (pI > 8). The average protein instability index is 53.57, with a distribution range of 36.44–65.43. Among them, ZoDUF668-7 is a stable protein, while the rest are unstable proteins (instability index > 40). The hydrophilicity (GRAVY) of ZoDUF668 proteins varied from −0.598 to −0.12, suggesting that they are hydrophilic proteins (hydropathicity < 0). The subcellular localization prediction results show that 50% of ZoDUF668 proteins are located in chloroplasts, 42% of ZoDUF668 proteins are located in the nucleus, and only ZoDUF668-7 is located in the cell membrane.

**Figure 3:**
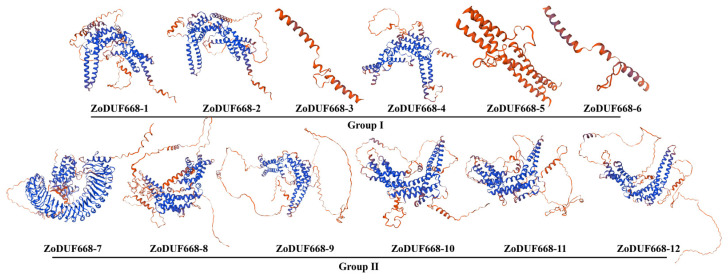
The 3D modeling of ZoDUF668 proteins.

The three-dimensional structure prediction results indicate that the ZoDUF668 protein is mainly composed of *α*-helix, random coil, extended strand, and *β*-turn (Figure 3), with *α*-helix accounting for the largest proportion (41.53–64.54%), with an average value of 52.33%; Next is the random coil (23.99–39.14%), with an average value of 30.48%, followed by extended strand (6.79–16.46%), with an average of 10.91%; The proportion of *β*-turn is the smallest (3.85–9.66%), with an average of 6.27%.

### 2.4. Gene Structure and Conservative Motif Analysis

**Figure 4:**
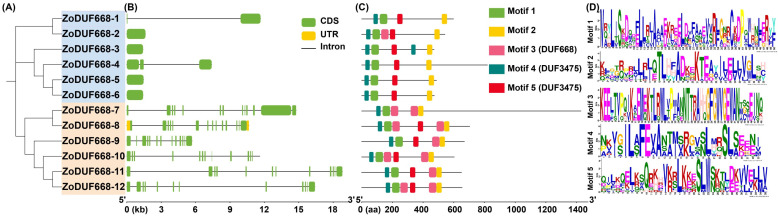
Gene structure and conservative motif analysis. (**A**) Phylogenetic tree of ZoDUF668s, different colored represent different species. (**B**) Gene structure analysis of ZoDUF668s, the green boxes, yellow boxes, and blacklines represent the exon, untranslated regions, and intron, respectively. (**C**) Motifs identification of ZoDUF668s. (**D**) Conserved amino acid sequences and functional domain of 5 motifs in ZoDUF668s. The seqlogo diagram shows that the conservatism of motifs at each position, with higher letters indicating better conservatism at that position. Different amino acids in the same position will be scaled according to their frequency.

Based on the annotation information provided in the GFF3 file, the *ZoDUF668* exon/intron structures in the ginger genome were visualized by TBtools. As shown in Figure 4, except for *ZoDUF668-2*, *ZoDUF668-3*, *ZoDUF668-5*, and *ZoDUF668-6*, and all *ZoDUF668s* contain 1–13 introns. *ZoDUF668-8* contains both 5′ and 3′ UTR regions, and the remaining *ZoDUF668s* contain no UTR regions. Conservative motif analysis showed that the distribution of the five motifs of the ZoDUF668 protein varies among different subfamilies, with each ZoDUF668 containing 4–6 conserved motifs (Figure 4C). Among them, Motif 4 and Motif 5 both constitute the key functional domains DUF347; Motif 3 constitutes DUF668 functional domain; and the other motifs do not match any known functional domain. Furthermore, all 12 ZoDUF668 genes contain Motif 1, Motif 2, and Motif 4, indicating that Motif 1, Motif 2, and Motif 4 exhibit high conservatism during evolution.

### 2.5. Promoter Analysis of ZoDUF668s

**Figure 5:**
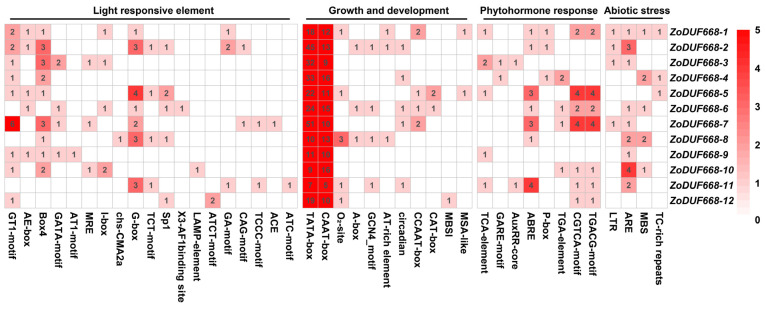
Analysis of *cis*-elements in promoter regions of *ZoDUF668* genes.

Plant CARE was used to predict the 1500 bp upstream promoter sequence of the *ZoDUF668* genes. The results showed that 42 types of *cis*-acting regulatory elements were identified from the promoter regions of *ZoDUF668* genes (Figure 5). These *cis*-elements can be divided into four groups. Among them, 19 *cis*-acting elements belong to “light-responsive elements”, 11 belong to “growth and development”, eight belong to “plant hormone response”, and four belong to “abiotic stress”. A large number of basic *cis*-acting elements, including TATA-box and CAAT-box, were commonly present in each ZoDUF668 promoter, implying that the ZoDUF668 gene plays an important role in the regulation of ginger growth and development. In addition, there are 19 kinds of light-responsive elements, including GT1-motif, AE-box, Box4, GATA-motif, I-box, G-box, and Sp1. Plant hormone response, including methyl jasmonate (TGACG-motif, CGTCA-motif), auxin (TGA-element, AuxRR-core), gibberellins (P-box, GARE-motif), abscisic acid (ABRE), and salicylic acid (TCA-element), were also detected in the *ZoDUF668* promoters. In addition, abiotic response elements, low-temperature response elements (LTR), oxidative stress response elements (ARE), drought-induced response elements (MBS), and defense and stress response elements (TC-rich repeats) were also present in the *ZoDUF668* promoter region, suggesting that *ZoDUF668s* may play a key regulatory role in stress response.

### 2.6. Expression Profile of ZoDUF668 Gene under Multiple Growth Stages and Stress Conditions

**Figure 6:**
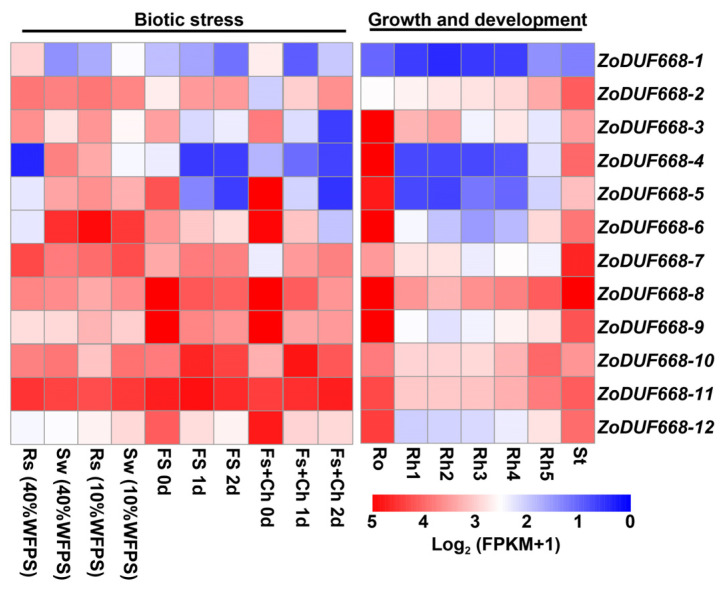
Expression profiles of the *ZoDUF668* gene under multiple growth stages, and *Fusarium solani* and *Ralstonia solanacearum* infection. St, aerial stem and leaves; Ro, roots; Rh1 to Rh5, rhizomes collected at five developmental stages based on their growth segments. Rs, *Ralstonia solanacearum*; Fs, *Fusarium solani*; WFPS, water-filled pore space; Ch, chitosan.

The expression patterns of the *ZoDUF668* gene at different growth stages and stress were analyzed using the RNA-seq data of ginger. As shown in Figure 6, *ZoDUF668s* respond to various biotic stresses. For instance *Fusarium solani* and *Ralstonia solanacearum* infection, *ZoDUF668-2*, *ZoDUF668-7*, *ZoDUF668-10*, and *ZoDUF668-11* were highly expressed under *F. oxysporum* after 1 d, while *ZoDUF668-1*, *ZoDUF668-5*, *ZoDUF668-6*, *ZoDUF668-8*, *ZoDUF668-9*, and *ZoDUF668-12* rapidly decreased with the increase in inoculation time. Under *F. solani* and chitosan, *ZoDUF668-2*, *ZoDUF668-7*, and *ZoDUF668-11* increased with the increase in inoculation time; *ZoDUF668-1*, *ZoDUF668-3*, *ZoDUF668-7*, *ZoDUF668-8*, and *ZoDUF668-11* were highly expressed under *R. solanacearum* infection (40% water-filled pore space), and *ZoDUF668-2*, *ZoDUF668-5*, *ZoDUF668-6*, and *ZoDUF668-8* were highly expressed under *R. solanacearum* treatment (10% water-filled pore space).

*ZoDUF668s* participate in the growth and development, with expression in roots and aerial stem and leaves higher than in rhizomes. *ZoDUF668-2*, *ZoDUF668-8*, *ZoDUF668-10*, *ZoDUF668-11*, and *ZoDUF668-12* show increased expression levels as the aerial stem leaves grow, while *ZoDUF668-3*, *ZoDUF668-7*, and *ZoDUF668-11* show decreased expression levels as the aerial stem leaves grow.

### 2.7. Post-Transcription Regulation of miRNA to ZoDUF668s

**Figure 7:**
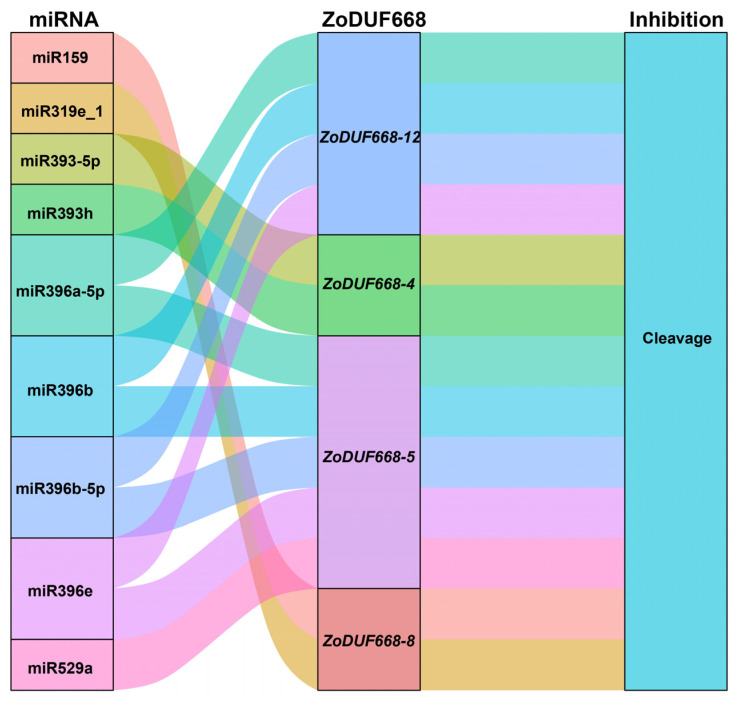
Sankey diagram of miRNA targeting relationship with *ZoDUF668s*. The three columns represent miRNA, mRNA, and inhibition effect.

As can be seen from Figure 7, all nine miRNAs target four *ZoDUF66s* genes through the cleavage effect. Notably, *ZoDUF668-8* was potentially targeted by two miRNAs, including miR159 and miR319e_1; miR529a regulates the expression of *ZoDUF668-5*; and miR396a_5p, miR396b, miR396b_5p and miR396e were predicted to target to *ZoDUF668-5* and *ZoDUF668-12* simultaneously. These results indicate that multiple miRNAs target *ZoDUF668s* and form a miRNA-mRNA regulatory network, participating in the post-transcriptional expression regulation of *ZoDUF668s*.

### 2.8. GO Function Enrichment Analysis

**Figure 8:**
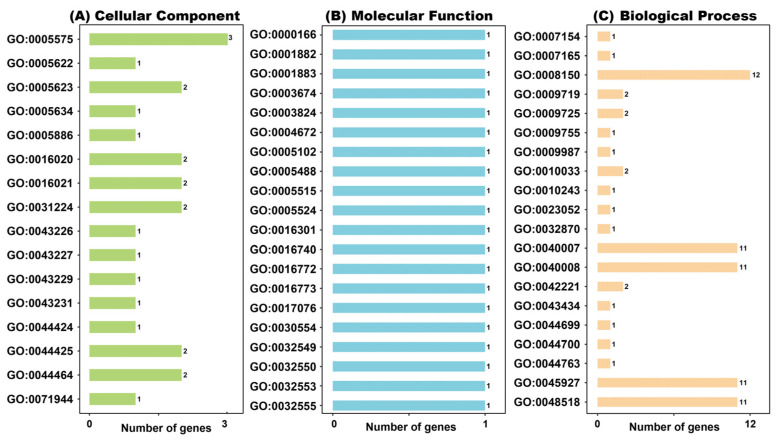
Gene Ontology (GO) classification based on ZoDUF668 annotation. GO terms are divided into three main categories, orange represents cellular components, blue represents molecular functions, and orange represents biological processes.

GO (Gene Ontology) analysis of the ZoDUF668 protein (Figure 8) showed that the biological pathways that the ZoDUF668 protein mainly participates in include cellular processes (3 ZoDUF668s), molecular functions (ZoDUF668-7), and single biological processes (12 ZoDUF668s). These ZoDUF668 proteins constitute cellular components, such as cells, organelles, membrane and so on. It is worth noting only ZoDUF668-7 exerts molecular function, including nucleotide binding (GO:0000166, GO:0001882), ATP binding (GO:0005524), and transferase activity (GO:0016740). In addition, the molecular functions of “response to stimulus (GO:0050896)”, “positive regulation of growth (GO:0045927)”, and “biological regulation (GO:0065007)” provide evidence of the involvement of ZoDUF668 in ginger growth and development, as well as its response to environmental stressors.

### 2.9. ZoDUF668s Respond to Biotic Stress

**Figure 9:**
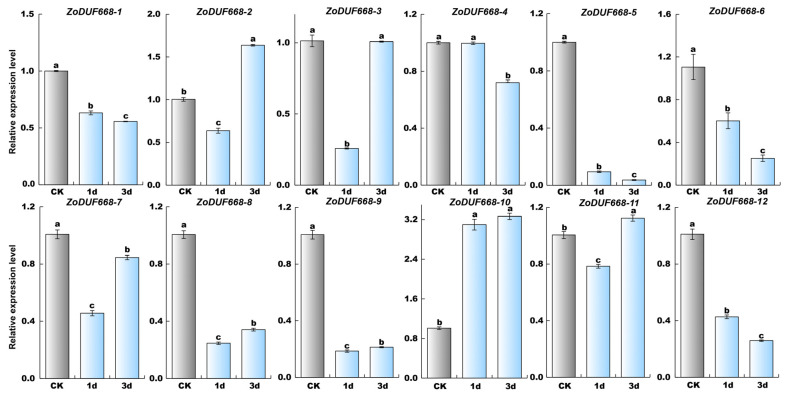
Analysis of *ZoDUF668* expression under *F*. *solani* stress. The *y* axis represents the relative expression level, and the *x* axis represents the time point after stress treatment. Each bar represents the average of nine replicates, and error bar represents standard deviation (SD), *n* = 9. Different letters in columns indicate significant differences between the treatments at *p* < 0.05 level.

To obtain a comprehensive comprehension of the functional significance of *ZoDUF668s* in response to biotic stress, the expression patterns of 12 *ZoDUF668s* were analyzed using quantitative RT-qPCR in response to *F. solani* stress (Figure 9). The results showed that the expression levels of *ZoDUF668-5*, *-6*, and *-12* continuously decreased within 3 days after treatment with *F*. *solani*. Among them, *ZoDUF668-5* exhibited an expression level of only 4% compared to the CK at 3 days post-treatment. In contrast, the expression level of *ZoDUF668-10* increased continuously over time, reaching 3.2-fold higher than the CK at 3 days post-treatment.

### 2.10. ZoDUF668s Respond to Abiotic Stress

**Figure 10:**
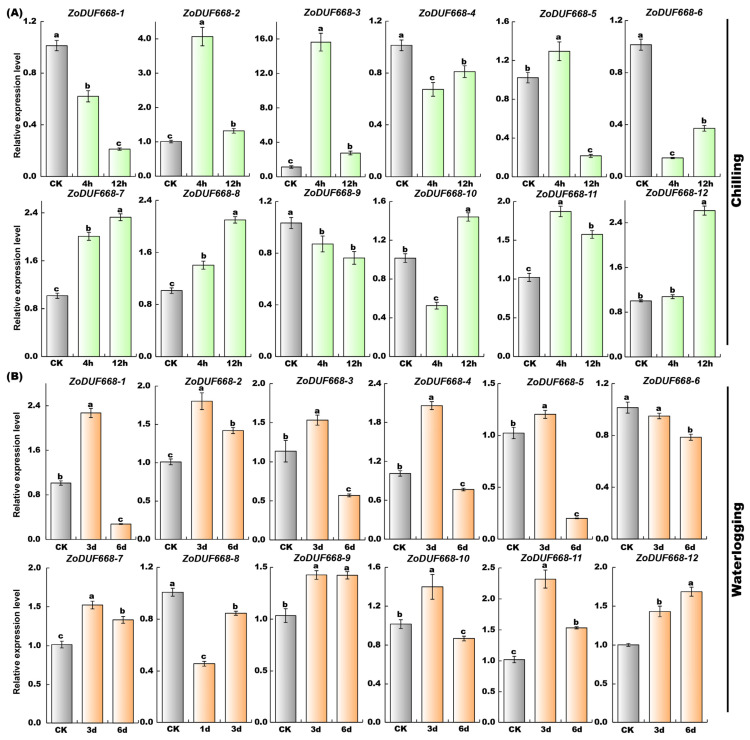
Analysis of *ZoDUF668* expression under chilling and waterlogging stresses. (**A**) Chilling and (**B**) waterlogging stress. The *y* axis represents the relative expression level, and the *x* axis represents the time point after stress treatment. Each bar represents the average of nine replicates, and error bar represents standard deviation (SD), *n* = 9. Different letters in columns indicate significant differences between the treatments at *p* < 0.05 level.

As illustrated in Figure 10, *ZoDUF668s* play an implant role in plants respond to abiotic stress. Under chilling injury, the expression levels of *ZoDUF668-2*, *-3*, and *-11* significantly increased after 4 h of treatment, among them, *ZoDUF668-3* showed the most significant increase, reaching 13.8-fold higher than the CK; subsequently, the expression levels decreased after 12 h but remained higher than the CK. The expression levels of *ZoDUF668-7* and *-8* continued to increase within 12 h of treatment, and were 2.3- and 2.1-fold higher than CK at 12 h of treatment, respectively.

Under waterlogging stress, the expression levels of 7 *ZoDUF668s* showed an initial increase followed by a decrease. However, the expression level of *ZoDUF668-12* continued to increase within 6 days of treatment and was 1.7-fold higher than the CK at 6 days. Additionally, *ZoDUF668-9* showed an initial increase in expression level, reaching 1.5 times higher than the control at 3 days and remained stable thereafter.

## 3. Discussion

Ginger is an important spice crop, and its cultivation process is susceptible to various biotic and abiotic stresses [14]. Relevant research shows that that the DUF668 gene plays an important role in plant growth and development and stress responses [6]. Despite this, the classification, evolution, and function of this gene family in ginger have not been systematically studied. In this study, 12 *ZoDUF668s* were identified from the ginger genome and separated into two subfamilies according to the phylogenetic tree branches and designated as *ZoDUF668-1* to *ZoDUF668-12*.

Structure determines function, and the structural diversity of exons/introns often plays a key role in the evolution of gene families [15]. The gene structure result shows that there are more introns of ZoDUF668 in Group II than in Group I. This phenomenon, with one set of DUF668 introns significantly higher than the other, is consistent across various plants such as wheat [10], rice [3], and cotton [6]. The difference in the number of introns may be one of the reasons for the functional differentiation of *ZoDUF668s* [16].

The *cis*-elements in the promoter region participate in plant growth and development and respond to environmental changes by regulating the expression of related genes [17]. There was a significant positive correlation between upstream promoter response genes and their *cis*-elements [18]. Previous studies have shown that *cis*-elements, such as AE-box, ATCT-motif, G-box, GT1-motif, GATA-motif, Box-4, AT-rich, and I-box, are important for the regulation of light-mediated transcriptional activity [19]. In this study, we found the G-box, Box-4, and GT1 motifs are the most abundant elements in ginger *ZoDUF668s*; simultaneously, these *cis*-elements have been identified in the study of *Ipomoea batatas* [13] and *Triticum aestivum* [10], strongly implying that, through these light-responsive *cis*-elements, *ZoDUF668s* play a role in photosynthesis and therefore regulate gene expression. Furthermore, cis-elements in response to abiotic stress, such as LTR in response to low temperature, ARE related to hypoxia stress, MBS related to drought stress and TC-rich in response to defense and stress, were also detected in the promoter region of *ZoDUF668s*. RT-qPCR analysis revealed that the expression levels of 6 *ZoDUF668s* increased under cold stress, while the expression levels of 10 *ZoDUF668s* increased within 3 days of waterlogging stress. Therefore, we speculate that when plants are subjected to chilling and waterlogging stresses, the *cis*-elements in the promoter region upregulate the expression of *ZoDUF668s* in response to these stresses.

Research has indicated that *DUF668* plays a significant role in plant responses to biotic stresses. In *G. hirsutum*, *GhDUF668-05/08/11/23/28* were significantly upregulated under drought and *Verticillium dahliae*, thus participating in the stress response process of cotton [6]. In *O. sativa*, the expression levels of *OsDUF668-1*/*4*/*5* are significantly upregulated under the pathogen stress of rice blast fungus, while the expression levels of *OsDUF668-6*/*7*/*11*/*12* are significantly decreased [3]. Similarly, the expression levels of *ZoDUF668s* are also regulated by the pathogen stress of *R. solanacearum* and *F. solani*. Meanwhile RT-qPCR analysis revealed that three genes (*ZoDUF688-2*, *-10*, and *-11*) were upregulated under *F. solani stress*. Among them, *ZoDUF668-10* showed significantly higher expression than the control (CK) within 3 days of treatment, implying *ZoDUF668-10* may be a resistance gene. Therefore, in the subsequent study, we will construct an overexpression vector for *ZoDUF668-10* to further validate its role as a disease-resistant gene and elucidate its biological function.

There are two main mechanisms by which miRNAs exert their effects. One class of miRNAs exhibits complementarity to their target mRNA and interferes with gene expression through a cleavage activity, leading to degradation of the target mRNA; the other class of miRNA functions by inhibiting the translation process, thereby regulating gene expression at the post-transcriptional level [20]. Previous studies have shown that miR159 can enhance plant tolerance to abiotic stress [21], such as in *G. max*, where the expression of miR159 decreases under cold stress [22]. In this study, we observed that the expression level of *ZoDUF668-8*, which is targeted by miR159, specifically increased under chilling stress conditions. Based on these findings, we speculate that in ginger, chilling stress leads to a downregulation of miR159 expression, consequently resulting in an upregulation of *ZoDUF668-8* expression. In addition, as most members of the miR396 family showed downregulation in expression levels under waterlogging stress in *Cucumis sativus* [23], and combined with RT-qPCR analysis, we speculate that waterlogging stress in ginger would also lead to a decrease in the expression levels of miR396, resulting in an upregulation of the expression levels of *ZoDUF668-5* and *ZoDUF668-12* to respond to the waterlogging stress.

## 4. Materials and Methods

### 4.1. Identification and Phylogenetic Analysis of ZoDUF668 Gene

The hidden Markov model file of the DUF668 protein (PF05003) was downloaded from Pfam database (http://pfam.xfam.org/, accessed on 15 October 2023) [24]. Then, the protein sequence database of ginger was searched by BLASTp (e-value < 1 × 10^−5^) to obtain the candidate protein for ZoDUF668. The DUF668 protein domain of ginger was further identified using Pfam (v35.0, http://Pfam.xfam.org/, accessed on 15 October 2023) and InterProScan (v94.0, http://www.ebi.ac.uk/InterProScan/, accessed on 15 October 2023) databases, eliminating repetitive, redundant, and incompletely annotated sequences [25]. The ClusterW multiple sequence alignment was performed on six *Arabidopsis thalian* [3], 12 *Oryza sativa* [3], 14 *Zea mays* [3], and 99 Gossypium [6] DUF668 members using iTOL online tools (Interactive Tree of Life, http://ITOL.embl.de, accessed on 15 October 2023) [26]. Phylogenetic trees, including *Z. officinale*, *A. thaliana*, *O. sativa*, *Z. mays*, and Gossypium, were constructed through the neighbor-joining method (repeat value set to 1000) to illustrate the phylogenetic relationship between DUF668s and members of ZoDUF668s were classified and named based on the phylogenetic relationship [25].

### 4.2. Chromosome Location Analysis

The chromosome locations of *ZoDUF668s* are extracted from the ginger genome GFF file, according to the start and end position information of *ZoDUF668s* on the chromosome, and the chromosome distribution map is drawn with “MapInspect” [9].

### 4.3. Gene Structure and Conserved Motifs Analysis of the ZoDUF668 Gene Family

According to GFF3 gene structure annotation information, the gene exon/intron structure map of *ZoDUF668s* was drawn using TBtools [9]. Conservative motifs were analyzed using MEME (https://meme-suite.org/meme/, accessed on 15 October 2023), parameter setting: the width of motifs ranged from 6 to 50 aa; any number of repetitions; the maximum fundamental number was 5. Then, the conserved motifs were visualized by TBtools [27].

### 4.4. Prediction of ZoDUF668 Physical and Chemical Properties

Analysis of the amino acid sequence of ZoDUF668, including protein length, isoelectric point (pI), molecular weight (MW), average value of stability, and grand average of hydrophilicity (GRAVY), was performed using the ExPASy (https://prosite.expasy.org/PS50011, accessed on 17 October 2023) online tool [28]. Prediction the secondary configuration of proteins by SOPMA (https://npsa-prabi.ibcp.fr/cgi-bin/npsa_automat.pl?page=npsa_sopma.html, accessed on 17 October 2023) online tool, and the three dimensional homology modeling was predicted with SWISS-MODEL (https://www.swissmodel.expasy.org/, accessed on 17 October 2023) [29]. The signal peptide prediction was performed using SignalP 5.0 (http://www.cbs.dtu.dk/services/SignalP/, accessed on 17 October 2023), and the subcellular localizations of ZoDUF668s were predicted by Plant-mPLoc (http://www.csbio.sjtu.edu.cn/bioinf/plant-multi/?tdsourcetag=s_pcqq_aiomsg, accessed on 17 October 2023) online tool [30].

### 4.5. Cis-Element Analysis

For *cis*-acting element analysis, the upstream promoter sequence (1–1500 bp) of *ZoDUF668* was manually extracted from the genome sequence and uploaded into the PlantCARE database to identify *cis*-elements in the promoter region [31]. The R software package “pheatmap” (R-4.2.2) was used to organize and display the analysis results.

### 4.6. Transcriptomic Analysis of ZoDUF668s

Ginger RNA-seq data were downloaded from the NCBI database and mapped to ginger genome using Hisat2 [32]. The expression level of *ZoDUF668s* (Fragments Per Kilobase of exon model per million mapped fragments) was calculated by Cufflinks. Log_2_ (FPKM + 1) values and R package “pheatmap” (R-4.2.2) were used to generate the heatmap to illustrate the expression profiling of *ZoDUF668s* under various growth stages and stress conditions [33].

### 4.7. Prediction of miRNAs Targeting Relationship

The mature coding sequences of ginger miRNAs were collected from the previous studies, the coding sequence and miRNA sequences were uploaded to psRNATarget (https://www.zhaolab.org/psRNATarget/, accessed on 22 October 2023) database [34]. Then, the mapping of the targeting relationship between miRNAs and *ZoDUF668s* was performed using the “ggalluvia” package in R software (R-4.2.2) [30].

### 4.8. Gene Ontology (GO) Data Analysis and Prediction of ZoDUF668s

The GO (Gene Ontology) database was used for functional annotation of the ZoDUF668 gene. GO (Gene ontology) analysis is performed in python. The full-length amino acid sequence of ZoDUF668 proteins was uploaded to the original program, and then drawn and annotated [35]. The analysis results provide a three-layer structure definition method for ZoDUF668 protein, which are biological process (BP), cellular component (CC), and molecular function (molecular function, MF), used to describe the related functions of ZoDUF668 protein.

### 4.9. Plant Materials

Ginger (“Fengtoujiang”, Jingzhou, Hubei Province, China) is cultivated in a glass greenhouse at the college of Horticulture and Gardening, Yangtze University. Healthy and uniformly sized ginger seeds were selected for disinfection and germination, and then planted directly (the temperature was set to 26 °C, relative humidity of 70~80% with 12 h/12 h day/night period). When the plant reached a height of about 90 cm, seedlings with the same growth were selected for stress treatment. The different treatments were as follows: (a) inoculation with *Fusarium solani*, all tested strains were provided by the Xiangxin Crop Research Institute of Yangtze University. It was cultured on a PDA plate for 3–4 d under dark conditions of 28 °C. The spores were collected by rinsing the culture medium with sterile water, and the concentration of spore suspension was adjusted to 1 × 10^4^ sporangia ml^−1^. The ginger seedling basal part of stems were uniformly wounded using a needle prick method and then injected with 30 μL of spore suspension, with sterile water treatment as the CK control. The infection sites were collected 1 and 3 d after inoculation with *F. solani* and immediately frozen in liquid nitrogen. After (c) chilling (10 °C) for 0, 4 and 12 h of treatment, the leaves were collected and immediately frozen in liquid nitrogen. The samples treated for 0 h were used as CK control. After (d) waterlogging (water submerged 1 to 2 cm above the rhizome of ginger) for 0, 3 and 6 d of treatment, the roots were collected. The samples treated for 0 h were used as CK control [36]. The collected tissue samples will be stored at temperature of −80 °C until further use. For each treatment, there were three biological replications and three technical replications, and at least ten plants were planted.

### 4.10. RNA Extraction and Quantitative Real-Time PCR Analysis

Total RNA extracted from collected ginger tissue using TRIzol reagent (GenStar, Beijing, China), and then reverse transcribe the total RNA into cDNA using HiScript II 1st Strand cDNA Synthesis Kit (+gDNA wiper) (Vazyme, Nanjing, China). Each treated cDNA sample was performed using ChamQ SYBR qPCR Master Mix (Vazyme, Nanjing, China) on the CFX 96 Real-Time PCR system (Bio-Rad, Hercules, CA, USA). The reaction system (20 μL) is as follows: 10 μL 2 × ChamQ SYBR qPCR Master Mix, forward and reverse primers each 0.4 μL, cDNA 2 μL, add ddH_2_O to 20 μL. The reaction procedure was as follows: 95 °C for 3 min, 95 °C for 30 s, 55 °C for 30 s, 72 °C for 20 s, a total of 35 cycles. The relative expression level of *ZoDUF668s* was calculated using the 2^−ΔΔCt^ method [37]. Each treatment includes three biological replications, and three technical duplications were performed to each cDNA.

### 4.11. Statistical Analysis

Excel 2021 was used to sort out and analyze the data, and student’s *t*-test was used for difference significance analysis.

## 5. Conclusions

In this study, a total of 12 DUF668 family members were identified from the ginger genome, which were located on six chromosomes, and divided into two subfamilies according to evolutionary relationships. All of them were hydrophobic proteins and unstable proteins. The *ZoDUF668* genes may be involved in the regulation of biotic and abiotic stress responses of ginger, including *F. solani*, waterlogging, and chilling stresses. This study laid a foundation for elucidating the function of *ZoDUF668* genes in ginger.

## Figures and Tables

**Table 1:** Characterization of ZoDUF668 protein.

Name	Gene ID	Len (aa)	MW (kDa)	pI	Atoms	Ins	Sta.	GRAVY	Sub.
ZoDUF668-1	Maker00038732	598	67.18	8.19	9461	53.19	unstable	−0.153	Nucleus
ZoDUF668-2	Maker00008971	542	60.70	7.55	8536	56.39	unstable	−0.151	Nucleus
ZoDUF668-3	Maker00015846	471	52.91	8.99	7477	61.19	unstable	−0.325	Chloroplast
ZoDUF668-4	Maker00022604	819	91.12	9.15	12,756	65.43	unstable	−0.351	Nucleus
ZoDUF668-5	Maker00068009	487	54.85	9.59	7777	61.04	unstable	−0.29	Chloroplast
ZoDUF668-6	Maker00077672	473	53.11	9.68	7508	63.83	unstable	−0.269	Chloroplast
ZoDUF668-7	Maker00061506	1428	156.83	6.64	22,128	36.44	stable	−0.12	Cell membrane
ZoDUF668-8	Maker00022935	703	79.57	8.57	11,217	50.84	unstable	−0.464	Chloroplast
ZoDUF668-9	Maker00055763	669	74.67	9.32	10,544	48.28	unstable	−0.456	Chloroplast
ZoDUF668-10	Maker00078796	603	67.72	9.13	9586	44.7	unstable	−0.431	Chloroplast
ZoDUF668-11	Maker00034739	650	72.14	9.07	10,148	55.51	unstable	−0.59	Nucleus
ZoDUF668-12	Maker00078495	654	72.53	9.37	10,205	46.08	unstable	−0.598	Nucleus

Note: Len, length of amino acid (aa); MW, molecular weight; pI, theoretical pI; Atoms, total number of atoms; Ins. index, instability index; Sta., Stability; GRAVY, the grand average of hydropathicity; Sub., predicted subcellular location.

## Data Availability

Data is contained within the article.
